# Association of dyslipidemia with the severity and mortality of coronavirus disease 2019 (COVID-19): a meta-analysis

**DOI:** 10.1186/s12985-021-01604-1

**Published:** 2021-07-27

**Authors:** Yanli Liu, Yilong Pan, Yuyao Yin, Wenhao Chen, Xiaodong Li

**Affiliations:** Department of Cardiology, Shengjing Hospital of China Medical University, 36 Sanhao Street, Shenyang, Liaoning 110004 People’s Republic of China

**Keywords:** Dyslipidemia, COVID-19, SARS-CoV-2, Severity, Mortality

## Abstract

**Background:**

The numbers of confirmed cases of coronavirus disease 2019 (COVID-19) and COVID-19 related deaths are still increasing, so it is very important to determine the risk factors of COVID-19. Dyslipidemia is a common complication in patients with COVID-19, but the association of dyslipidemia with the severity and mortality of COVID-19 is still unclear. The aim of this study is to analyze the potential association of dyslipidemia with the severity and mortality of COVID-19.

**Methods:**

We searched the PubMed, Embase, MEDLINE, and Cochrane Library databases for all relevant studies up to August 24, 2020. All the articles published were retrieved without language restriction. All analysis was performed using Stata 13.1 software and Mantel–Haenszel formula with fixed effects models was used to compare the differences between studies. The Newcastle Ottawa scale was used to assess the quality of the included studies.

**Results:**

Twenty-eight studies involving 12,995 COVID-19 patients were included in the meta-analysis, which was consisted of 26 cohort studies and 2 case–control studies. Dyslipidemia was associated with the severity of COVID-19 (odds ratio [OR] = 1.27, 95% confidence interval [CI] 1.11–1.44, P = 0.038, I^2^ = 39.8%). Further, patients with dyslipidemia had a 2.13-fold increased risk of death compared to patients without dyslipidemia (95% CI 1.84–2.47, P = 0.001, I^2^ = 66.4%).

**Conclusions:**

The results proved that dyslipidemia is associated with increased severity and mortality of COVID-19. Therefore, we should monitor blood lipids and administer active treatments in COVID-19 patients with dyslipidemia to reduce the severity and mortality.

**Supplementary Information:**

The online version contains supplementary material available at 10.1186/s12985-021-01604-1.

## Introduction

Coronavirus disease 2019 (COVID-19) swept the world by manifestation as the severe acute respiratory syndrome coronavirus 2 (SARS-CoV-2). This disease initially appeared in Wuhan, China in December 2019 and then spread rapidly worldwide in early 2020. On January 30, 2020, the World Health Organization (WHO) announced that COVID-19 was a public health emergency of international concern. Due to the continuous and rapid transmission of SARS-CoV-2, COVID-19 has caused a global health crisis that has drastically affected normal life, the economy, and politics. Although some preventative measures have been taken, the numbers of confirmed cases and deaths is still rising worldwide. On April 22, 2021, the WHO reported 143,184,614 confirmed cases and 3,047,322 deaths [1].

SARS-CoV-2 is the third highly pathogenic coronavirus found to infect humans, following the severe acute respiratory syndrome coronavirus in 2003 and the Middle East respiratory syndrome coronavirus in 2012 [2]. SARS-CoV-2 can be transmitted through respiratory droplets into the mucous membranes of the eyes or oral cavity and travel to important organs such as the heart and lungs. Patients infected with SARS-CoV-2 show different clinical symptoms, ranging from mild symptoms such as fever and cough to more serious symptoms such as bilateral interstitial pneumonia, and severe infection may cause acute respiratory distress syndrome and terminal organ failure [3].

Dyslipidemia is a common complication in patients with COVID-19. Reduction in total cholesterol and low-density lipoprotein cholesterol (LDL-C) in patients with COVID-19 has been reported [4, 5], indicating that there may be a pathophysiological interaction between lipid metabolism and vascular disease in the progression of COVID-19. However, the association between dyslipidemia and COVID-19 has not been established. Therefore, we conducted a meta-analysis of current studies to the possible interplay between dyslipidemia and the severity and mortality of COVID-19. This assessment will be helpful to further understand the characteristics of COVID-19 in patients with dyslipidemia.

## Materials and methods

### Study protocol and registration

This systematic review and meta-analysis was conducted in strict accordance with the Preferred Reporting Items for Systematic Reviews and Meta-Analyses guidelines [6]. The International Prospective Register of Systematic Reviews has reported this protocol (PROSPERO identifier: CRD42020222400).

### Search strategy

To discern high-quality evidence, we conducted a comprehensive search in a wide spectrum of databases, namely the PubMed, Embase, MEDLINE, and Cochrane Library databases for all relevant studies up to August 24, 2020. The search terms were as follows: “Corona Virus Disease-2019” or “2019 novel coronavirus” or “SARS-CoV-2” or “COVID-19” or “2019-ncov” or “new coronavirus pneumonia” and “hyperlipemia” or “hyperlipidemia” or “dyslipidemia” or “dyslipidaemia” or “hypolipidemia” or “clinical characteristics” or “comorbidities” or “risk factors” and “cohort” or “case control”. The retrieved studies were also manually searched to ensure other related studies.

### Selection criteria

The included studies were characterized as follows: (1) study design: case–control study or cohort study; (2) COVID-19 cases: all COVID-19 cases were confirmed on positive result of real-time reverse transcriptase–polymerase chain reaction for SARS-CoV-2 in swab samples and hospitalized; (3) parameters: COVID-19 patients with a history of dyslipidemia; (4) clinical outcomes: infection severity or mortality of COVID-19 reported; (5) "severe disease" was defined clearly; and (6) study sample size: at least 10 cases. In this analysis definition of severe disease was defined as COVID-19 confirmed patients with one of conditions: (1) arterial oxygen partial pressure/fraction of inspired oxygen ≤ 300 mmHg; (2) oxygen saturation ≤ 93% of indoor air at rest; (3) respiratory distress, respiratory rate ≥ 30 breaths/min; (4) patients requiring mechanical ventilation or vital life support or intensive care unit admission; (5) death. In this meta-analysis dyslipidemia included hyperlipidemia (hypertriglyceridemia or hypercholestromia) or hypolipidemia. When the information in the study was incomplete, we contacted the corresponding author to get the complete information.

### Outcomes of interest

The primary outcome was the relationship between dyslipidemia and the severity of COVID-19. The secondary outcome was the association between dyslipidemia and COVID-19 mortality. Therefore, two separate meta-analyses were conducted.

### Data extraction

Two authors (YLL and YLP) withdrew the useful data from all the included studies, and any disagreement was reconciled via discussion with the third author YYY. The following data were extracted from the included studies: (1) the name of the first author; (2) study type, study location, and study date; and (3) the general characteristics of the participants (age, body mass index and sample size in the non-severe cases, severe cases, death cases and survived cases).

### Quality assessment

The Newcastle Ottawa scale was used to assess the quality of the included studies, including COVID-19 case selection, comparability, and outcome of disease severity or mortality. Case selection included four items: (1) representativeness of cases; (2) appropriate determination of severe cases; (3) appropriate determination of non-severe cases; and (4) appropriate determination of blood lipid levels. The comparability factor assessed whether the comparability between patients with and without dyslipidemia was considered in the design and statistical analysis. The lowest score and the highest was 0 and 9 points, respectively. Studies with scores of 0 to 3 points, 4 to 6 points, and 7 to 9 points were considered to be of low, medium, and high quality, respectively [7].

### Data synthesis and analysis

All analysis was performed using Stata 13.1 software. Forest plots were employed to illustrate the severity and mortality of COVID-19 patients with and without dyslipidemia. The pooled odds ratio (OR) and 95% confidence interval (CI) were used to describe the statistical significance (P ≤ 0.05), which provided a quantitative estimate of the associations of dyslipidemia with the severity and mortality of COVID-19. The statistical heterogeneity of the included studies was expressed by I^2^, whose value is stratified as low (< 25%), moderate(25–50%), high(> 50%). Fixed effects models were used to compare the differences between studies. In this study, funnel plot and Egger’s test were used to evaluate the possibility of publication bias. A P value < 0.05 in the Egger’s test was considered to indicate publication bias.

## Results

### Search results

**Fig. 1:**
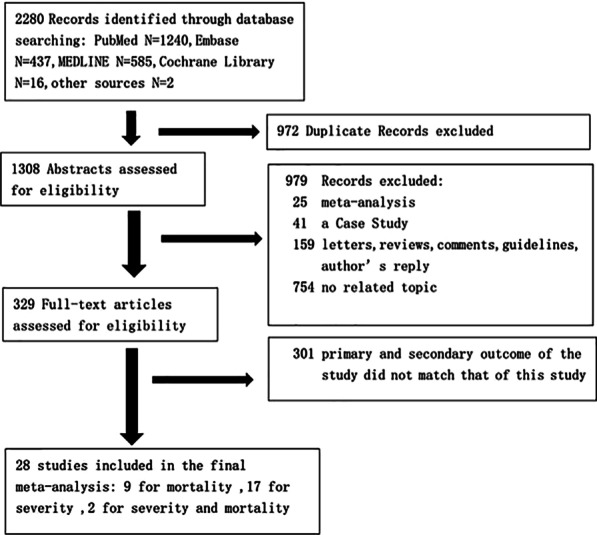
Flow chart showing the study selection

A total of 2280 studies were retrieved, of which 972 were duplicate studies. Nine hundred and seventy-nine studies were excluded after reading the titles and abstracts. Three hundred and one studies with inconsistent primary and secondary outcomes were excluded after reading the full text. Finally, 28 studies involving 12,995 patients with COVID-19 were included for this meta-analysis, including 9 studies on COVID-19 mortality risk, 17 studies on COVID-19 severity, and 2 studies on both severity and mortality [8–35]. The study selection flow diagram and exclusion reasons are shown in Fig. 1. All 28 included studies were published in 2020. The sample sizes ranged from 23 to 3988.

### Patient characteristics

The patient characteristics based on severity of COVID-19 are shown in Table 1. The patient characteristics based on mortality from COVID-19 are shown in Table 2.

**Table 1:** Patient characteristics based on COVID-19 severity

Study	Country (City)	Study design	Study period	Non-severe COVID-19				Severe COVID-19			
				Age (years)	BMI (kg/m^2^)	Dyslipidemia (n)	Non-dyslipidemia (n)	Age (years)	BMI (kg/m^2^)	Dyslipidemia (n)	Non-dyslipidemia (n)
Maeda [8]	USA (New York)	Cohort	Mar13–31, 2020	65 [51–77]	28.9 [24.7, 2.3]	64	103	67 [58–76]	27.2 [24.1, 31.8]	28	29
Ferguson [9]	USA (California)	Cohort	Mar13–Apr11, 2020	61.7 [46.6–72.9]	NA	17	34	57.6 [42.2–70.1]	NA	8	13
Zhang [10]	China (Wuhan)	Cohort	Jan16–Feb3, 2020	51.5 (26–78)	NA	5	77	64 (25–87)	NA	2	56
To [11]	China (Hong Kong)	Cohort	Jan22–Feb12, 2020	56 (37–75)	NA	0	13	66 (39–75)	NA	2	8
Rastrelli [12]	Italy (Mantua)	Cohort	NA	63.0 [55.0–66.5]	NA	5	16	NA	NA	3	7
Almazeedi [13]	Kuwait	Cohort	Feb24–Apr 20, 2020	37.1 ± 16	26.8 ± 7.7	58	996	54.8 ± 11	29.0 ± 5.1	7	35
Chen [14]	China (Zhejiang)	Cohort	Jan1–Mar11, 2020	45.3 ± 13.6	23.20 [21.66–25.71]	0	102	52.8 ± 15.5	24.78 [23.07–26.96]	1	42
Jurado [15]	Spain	Cohort	Second half of March 2020	NA	NA	105	286	NA	NA	54	108
Wu [16]	China (Wuhan)	Cohort	Dec23, 2019–Feb13, 2020	43.0 [33.0–61.0]	NA	11	217	62.0 [52.5–71.5]	NA	5	66
Urra [17]	Spain	Case- control	Mar1–Apr15, 2020	57.89 ± 13.1	NA	54	91	65.64 ± 14.1	NA	5	22
Gidari [18]	Italy (Perugia)	Cohort	Mar1–Apr20, 2020	63 ± 17	NA	8	33	65 ± 9	NA	2	25
Zhang [19]	China (Beijing)	Cohort	Jan–Feb 2020	45.34 ± 15.25	NA	2	54	64.75 ± 14.76	NA	1	23
de la Rica [20]	Spain (Mallorca)	Cohort	as of Mar 31, 2020	66.30 ± 14.90	NA	16	11	65.57 ± 12.87	NA	13	7
Simonnet [21]	France	Cohort	Feb27–Apr5, 2020	60 [50–72]	27 [25.3–30.8]	10	29	60 [51–69]	31.1 [27.3–37.5]	24	61
Petrilli [22]	USA (New York)	Cohort	Mar1–Apr8, 2020	60 [48–71]	NA	692	1047	68 [58–78]	NA	465	525
Li [23]	China (Wuhan)	Case- control	NA	51.45 ± 15.08	NA	48	376	61.54 ± 13.36	NA	8	91
Chang [24]	South Korea (Daegu)	Cohort	Feb28–Mar 31, 2020	36.47 ± 14.04	NA	4	194	54.08 ± 11.98	NA	2	11
Kong [25]	China (Wuhan)	Cohort	Jan27–Mar9, 2020	53.2 ± 15.6	NA	2	121	67.9 ± 12.3	NA	3	84
Lodigiani [26]	Italy (Milan)	Cohort	Feb13–Apr10, 2020	68 [55–77]	NA	69	258	61 [55–69]	NA	7	54

Data are expressed as mean ± standard deviation; median [interquartile range]; median (range)

*COVID-19* coronavirus disease, *NA* not available, *n* number, *BMI* body mass index

**Table 2:** Patient characteristics based on COVID-19 mortality

Author	Country (City)	Study design	Study period	Survived				Died			
				Age (years)	BMI (kg/m^2^)	Dyslipidemia (n)	Non-dyslipidemia (n)	Age (years)	BMI (kg/m^2^)	Dyslipidemia (n)	Non-dyslipidemia (n)
Almazeedi[13]	Kuwait	Cohort	Feb24–Apr20, 2020	38.7 ± 15.1	26.8 ± 5.9	62	1015	55.0 ± 10.1	33 ± 4.7	3	16
Khalil[27]	UK(London)	Cohort	Mar7–Apr7, 2020	63.8 (61.1–66.4)	NA	34	128	75.8 (72.5–79.2)	NA	16	42
Gayam[28]	USA(New York)	Cohort	Mar1–Apr9, 2020	63 [53–73]	28.3 [25.0–33.6]	41	235	71 [62–80]	31.8 [26.5–37]	25	107
Goicoechea[29]	Spain	Cohort	Mar12–Apr10, 2020	69 ± 14	27.2 ± 4.5	18	7	75 ± 6	25.1 ± 3.6	6	5
Santos[30]	Spain (Leon)	Cohort	Mar1–Jun1, 2020	75.1 [69.3–75.8]	NA	12	16	78.4 [74.5–83.5]	NA	9	1
Wang[31]	USA (New York)	Cohort	Mar1–Apr30, 2020	71 [18.5]	28.2 [10.2]	13	9	68 [8]	29.5 [9.9]	12	2
Ferrando[32]	Spain	Cohort	Mar12–May16, 2020	62 [53–71]	28.6 [25.7–32.4]	55	405	68 [62–73]	27.7 [25.3–31.6]	35	168
Zhang[19]	China (Beijing)	Cohort	Jan–Feb, 2020	NA	NA	2	75	84.00 ± 8.185	NA	1	2
Hwang[33]	South Korea (Daegu)	Cohort	Feb1–Mar25, 2020	64.62 ± 15.84	NA	10	67	76.50 ± 9.25	NA	3	23
Smith[34]	New England	Cohort	Mar1–Apr22, 2020	63.56	30.94	107	122	73.31	29.85	60	57
Grasselli[35]	Italy (Milan)	Cohort	Feb20–Apr22, 2020	NA	NA	169	1893	NA	NA	376	1550

Data are expressed as mean ± standard deviation; median [interquartile range]; mean (95%Confidence interval)

*COVID-19* coronavirus disease, *NA* not available, *n* number, *BMI* body mass index

### Data analysis

**Fig. 2:**
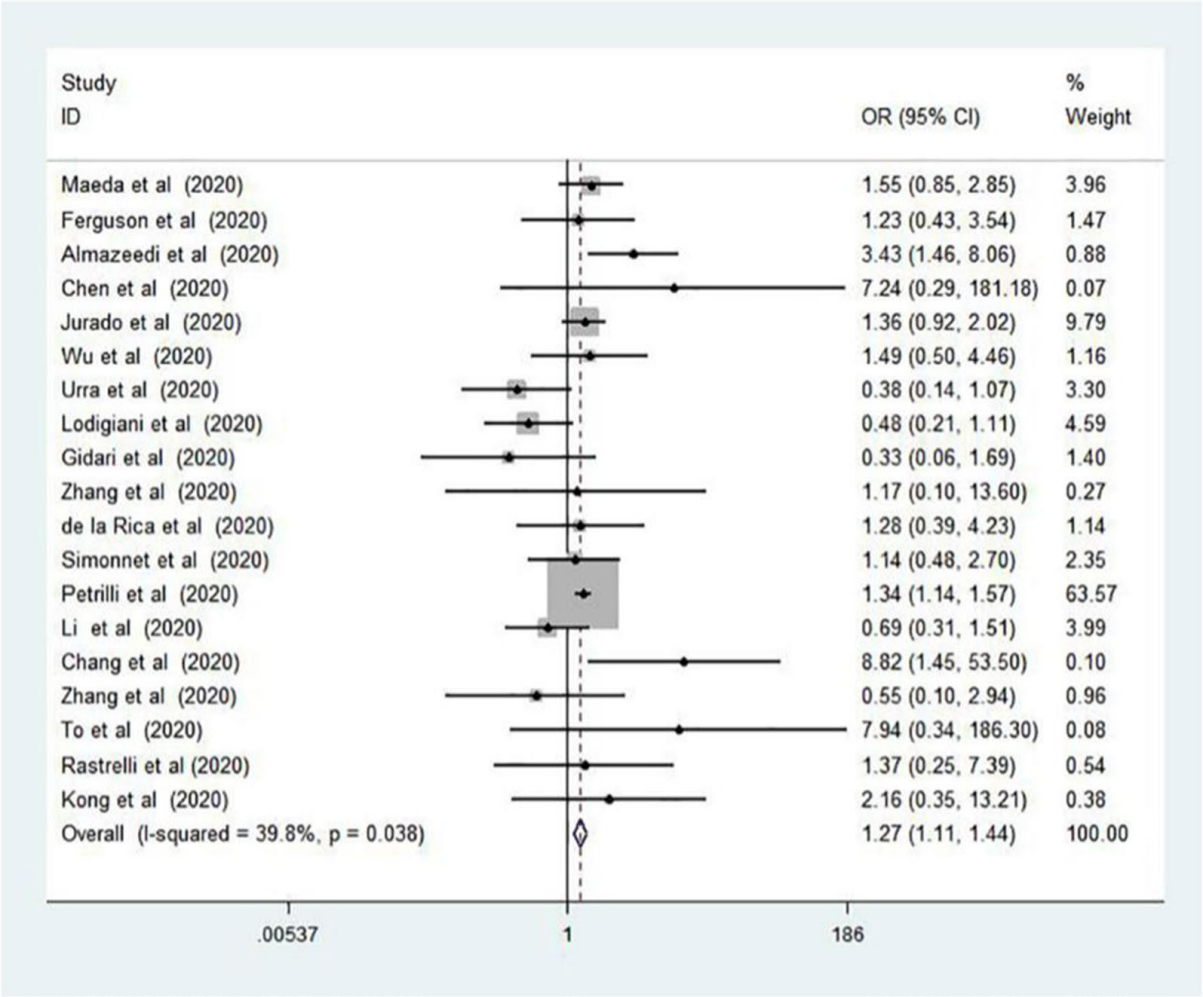
Forest plot demonstrating the association of dyslipidemia with COVID-19 severity. *COVID-19* coronavirus disease, *RR* risk ratio, *CI* confidence interval

**Fig. 3:**
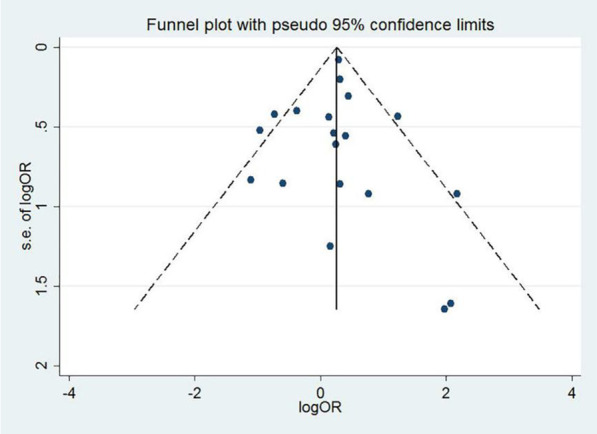
Funnel plot for the analysis of the association of dyslipidemia with COVID-19 severity. *COVID-19* coronavirus disease

**Fig. 4:**
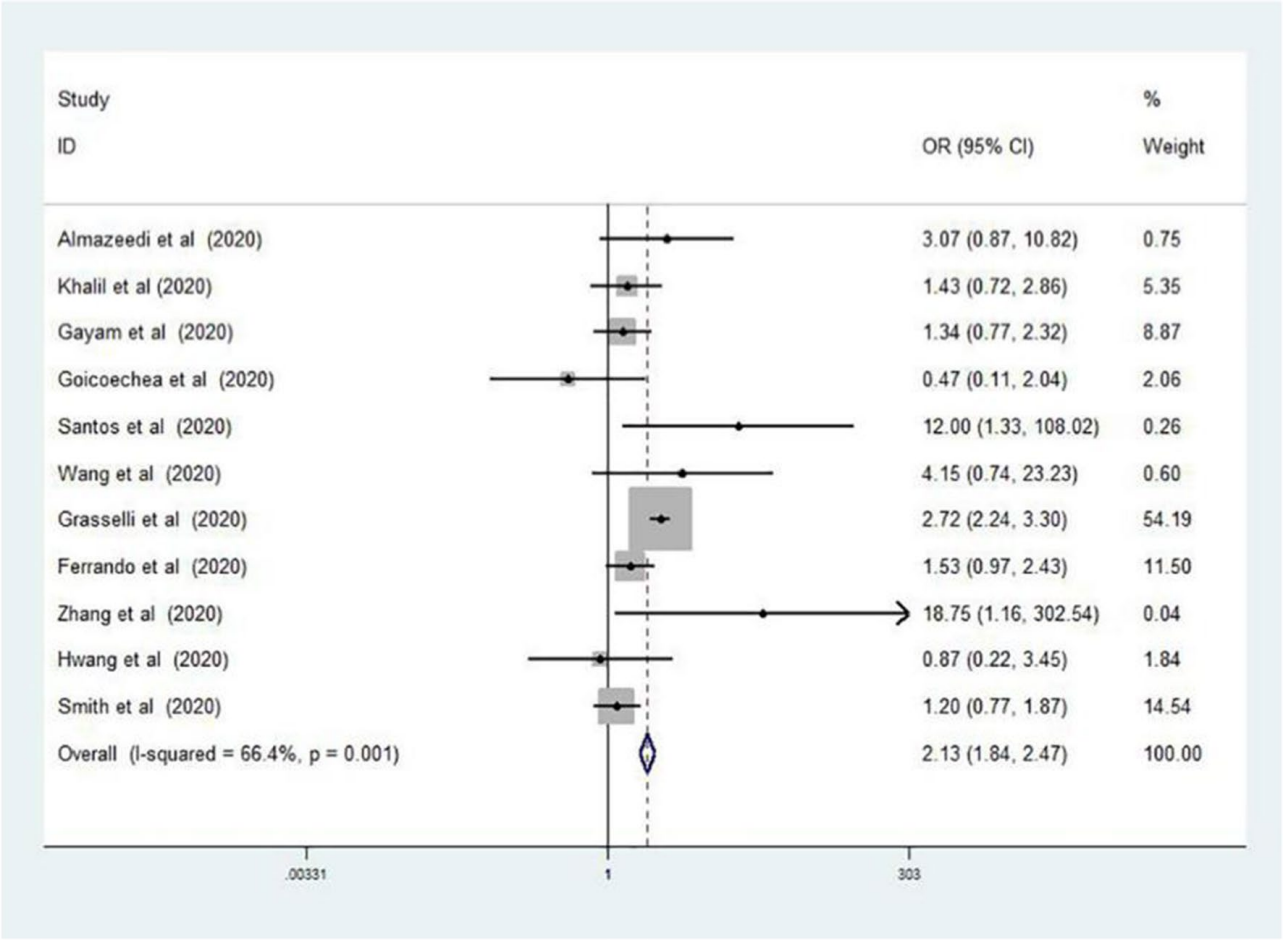
Forest plot demonstrating the association of dyslipidemia with COVID-19 mortality. *COVID-19* coronavirus disease; RR, risk ratio; CI, confidence interval

**Fig. 5:**
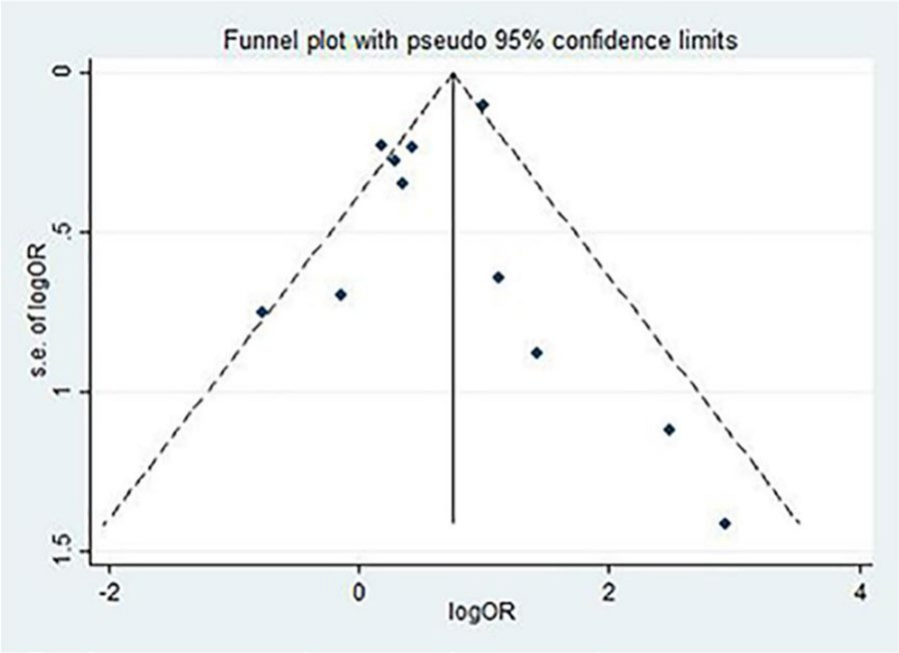
Funnel plot for the analysis of the association of dyslipidemia with COVID-19 mortality. *COVID-19* coronavirus disease

In the pooled analysis, dyslipidemia was significantly associated with the severity of COVID-19 (OR = 1.27, 95% CI 1.11–1.44, P = 0.038). In this analysis, moderate heterogeneity was observed (I^2^ = 39.8%; Fig. 2). There was no evidence of publication bias by Egger’s test (P = 0.856) or funnel plot (Fig. 3). In addition, compared to patients without dyslipidemia, patients with dyslipidemia had a higher risk of death (OR = 2.13, 95% CI 1.84–2.47, P = 0.001), and the heterogeneity was significant (I^2^ = 66.4%; Fig. 4). Then the sensitivity analysis demonstrating the association of dyslipidemia with COVID-19 mortality showed that the study [35] estimated OR was lower than 95% confidence interval lower limit (Additional file 3: Fig. S1). Get rid of this study, dyslipidemia was also associated with the mortality of COVID-19 (OR = 1.44, 95% CI 1.13–1.82, P = 0.153, I^2^ = 32.0%). Perhaps it was because there was unclear cut-off value for hypercholestromial in dyslipidemia. Similarly, Egger's test (P = 0.485) and funnel plot (Fig. 5) showed no potential publication bias in the analysis of mortality. The quality assessment of studies was shown in the Additional files 1, 2 Tables SI and SII.

## Discussion

This meta-analysis included 28 studies involving 12,995 patients with COVID-19. To our knowledge, this meta-analysis is based on the study that dyslipidemia is associated with severe COVID-19 infection by Hariyanto Ti et al. to assess the potential relationship between dyslipidemia and severity and mortality risk of COVID-19 [36].

Previous studies have shown that dyslipidemia is accountable for the increased severity and mortality of COVID-19, which is of great significance in clinical practice. Hypercholesterolemia is an independent risk factor of cardiovascular disease and chronic inflammation. An epidemiological study showed that total cholesterol (TC) is positively correlated with mortality in cardiovascular disease [37]. The LDL-C concentration in COVID-19 patients with dyslipidemia is high. With increased oxidative stress, low-density lipoprotein (LDL) forms oxidized LDL (oxLDL) after crossing the endothelial barrier. OxLDL can form immune complexes and contribute significantly to the initiation and transmission of inflammatory processes. OxLDL is also a powerful stimulator that can activate endothelial cells and monocytes, so it can increase the expression of a variety of inflammatory proteins and receptors [38]. OxLDL can also induce endothelial cell apoptosis through LDL receptor-1-mediated NF-κB signaling, caspase-3, caspase-9, and Fas, thus increasing monocyte levels, platelet activation, and vascular smooth muscle cell migration induced by collagen exposure [39–41]. The pathological results showed that the lung injury of COVID-19 patients was caused by endothelial cell pyroptosis and apoptosis [42]. Hyperlipidemia is an important factor leading to endothelial dysfunction. Lowering cholesterol levels will reduce the degree of vascular disease, thus protecting the integrity of endothelial cells from SARS-CoV-2. Further, the level of high-density lipoprotein cholesterol (HDL-C) is low in COVID-19 patients with dyslipidemia. High-density lipoprotein (HDL) can inhibit atherosclerosis through adenosine triphosphate binding cassette A1 [43]. HDL also induces cyclooxygenase-2, which is responsible for producing anti-inflammatory prostaglandins [44]. HDL even directly inhibits the expression of NF-κB in macrophages by inhibiting CD40 [45]. These chronic inflammatory states increase the risk of cardiovascular disease. HDL also is proved to be an anti-inflammatory lipoprotein [46]. It has been reported that inflammation leads to the structural changes of HDL granules and the accumulation of serum amyloid A (SAA). The HDL granules rich in SAA lose their anti-inflammatory properties. Recent the study showed that SAA levels increased dynamically with the severity of COVID-19 disease. SAA may be considered as a biomarker to evaluate the severity and prognosis of COVID-19 [47, 48]. In the experimental model, infusion of recombinant HDL reduced inflammation and the number of bacteria, alleviated organ damage and improved survival rate [49]. In the study C-reactive protein (CRP) levels were higher in patients with low HDL-C, which indicated that HDL-C may hamper inflammation and thus have a protective effect against COVID-19. Based on these, we speculate that HDL loses its anti-inflammatory properties and consumes cholesterol during COVID-19 infection. This may further explain the significant decrease of HDL-C concentration in patients with COVID-19 infection [50]. A retrospective study of 597 patients with COVID-19 demonstrated that patients with COVID-19 developed hypolipidemia at the earliest onset of mild symptoms. The decrease of LDL-C level was positively correlated with CRP level. Reduced lipid levels are likely the result of complex biological and pathological processes caused by SARS-CoV-2 infection [51]. Although LDL-C synthesis is increased, serum LDL-C decreases due to the simultaneous increase in LDL receptor expression [6]. This may be explained by the characteristic changes in the acute phase response seen in many other inflammatory processes, such as acute coronary syndromes [52]. In addition, some studies attributed this low lipid status to increased lipid exosmosis and liver synthetic dysfunction [51].

SARS-CoV-2 is a single-stranded RNA virus with an envelope and can enter host cells through endocytosis or membrane fusion. SARS-CoV-2 binds to ACE2 on the host cell membrane [53]. In addition, it has been suggested that SARS-CoV-2 leads to excessive activation of the immune system through activation of the Toll-like receptor-MyD88-NF-κB pathway, which induces a proinflammatory response, a so-called "cytokine storm," by releasing proinflammatory cytokines such as interferons, interleukins, and tumor necrosis factor [54]. This cytokine storm can cause severe organ damage. Furthermore, studies have indicated that endothelial cells of patients with herpes virus infection indicated thrombin formation. These patients also had increased adhesion of platelets and granulocytes [55, 56]. Span et al. studied endothelial cells infected with cytomegalovirus (CMV) and found that leukocytes and platelets adhere to these endothelial cells, and CMV can also increase cytokine expression [56, 57]. Dyslipidemia promotes the activation of interleukin 1 (IL-1) dependent pathway by activating nucleotide-binding oligomerization domain-, leucine-rich repeat- and pyrin domain-containing 3 (NLRP3) inflammasome, which is conducive to IL-1 cleavage and secretion, and modifie-LDL via scavenger receptors upregulates interleukin 6 (IL-6) in endothelial cells. Dyslipidemia promotes endothelial dysfunction and activation, which leads to the increase of pro-inflammatory cytokines and reactive oxygen species. These observations support that dyslipidemia promotes systemic inflammation by inducing multiple mediators [58–60]. Additionally, it is known that hyperlipidemia damages the immune response and may lead to persistent chronic inflammation, which leads to cardiovascular disease risk [61]. A meta-analysis suggested that an underlying or prior history of cardiovascular disease was directly associated with poor outcomes and severity in patients with COVID-19. Cardiovascular disease was found to be associated with an approximately three-fold increase in the risk of severe COVID-19 infection and an 11-fold increase in all-cause mortality in patients with COVID-19 [62]. Based on what has been observed in cardiovascular disease caused by dyslipidemia and chronic inflammation, it is possible that the SARS-CoV-2 acute inflammatory reaction aggravates the severity of the disease and increases the risk of mortality. Obesity may be key factors for COVID-19 in patients infected with SARS-CoV-2. The tissue expression of angiotensin converting enzyme 2 may play a important role in the disease development of COVID-19 obese patients, because the amount of adipose tissue in obese patients increases, resulting in an increase in the number of cells expressing angiotensin converting enzyme 2, thus increasing the risk of SARS-CoV-2 infection. Adipose tissue hypertrophy and hyperplasia promote triglyceride, phospholipid oxide, tumor necrosis factor-α, and adipocytokines contribute to obesit [63].

Lipids are the structural basis of both the cell membrane and the viral membrane, which play key roles in viral infection. Lipids are also involved in viral membrane fusion, encapsulation, and transformation. Lipid metabolism also plays an important role in the viral infection cycle, including virus entry, release, and replication, which may be prevented by changing the membrane lipid composition or lipid metabolism [64]. The lipid of virus is rich in cholesterol, so the consumption of cholesterol leads to decrease cholesterol content on virus membrane, and inhibit the infectivity of virus. In 2014, it was reported that statins, a limiting step in cholesterol biosynthesis, could inhibit the infectivity of Ebola virus disease [65]. Thus, hypolipidemic therapy has a dual beneficial effect in patients with hyperlipidemia by reducing the risk of cardiovascular disease and interfering with SARS-CoV-2 entry into cells. Therefore, patients with dyslipidemia should be advised to take preventive measures to minimize the risk of exposure to the virus. Patients with cardiovascular disease are at particular risk from COVID-19, as the infection will be more serious and the virus itself can cause cardiovascular damage. Thus dyslipidemia should be considered an important factor in the mortality of COVID-19. Additionally, reducing dietary cholesterol intake, increasing soluble fiber and soy protein intake and regular aerobic exercise, especially at least 120 min a week, have beneficial effects on blood lipid levels for reducing the risk of complications if COIVD is contracted [66].

As we know, this meta-analysis showed that dyslipidemia interconnects with the severity and mortality of COVID-19. However, the meta-analysis also has some limitations. First, unknown factors might interfere with the final result. Previous studies have shown that gender, age, nutritional status, smoking and diabetes can be implicated in the prognosis of patients with COVID-19, but other potential confounding factors were not excluded. In the future, the number of included studies will be increased, and it may be possible to analyze these potential confounding factors according to risk factors. Second, this meta-analysis only includes cohort studies and case–control studies. The lack of large-scale randomized controlled trials is the main limitation. In the future, more randomized controlled studies should be included. Third, hyperlipidemia and hypolipidemia were classified into dyslipidemia for analysis. None of the studies clearly indicated the accurate cut-off value of the diagnosis and definition of dyslipidemia, which may lead to error in the summary results and affect their accuracy. However, all included studies followed national or international guidelines for the diagnosis of dyslipidemia, and these studies were peer-reviewed. Fourth, the timing of the measurement of serum lipids and lipid-lowering drugs in each individual study were unclear, which may affect the true difference in severity and mortality of COVID-19 infection between patients with and without dyslipidemia. In addition, it is worth noting that funnel plots are a useful test for detecting bias in meta-analyses, but the ability to detect bias is limited when analyzing small trials. In the paper, most of the included studies were small, so the analysis should be treated with caution, which may affect the accuracy.

## Conclusion

In conclusion, the meta-analysis showed that dyslipidemia as a predictor might foretell the severity and mortality of COVID-19. Active management of blood lipids and monitoring of lipid levels may be beneficial for COVID-19 patients.

## Supplementary Information

**Additional file 1: Table SI**: Quality assessment of included studies (cohort studies)

**Additional file 2: Table SII**: Quality assessment of included studies (case–control studies)

**Additional file 3: Figure S1**: Sensitivity analysis demonstrating the association of dyslipidemia with COVID-19 mortality.

## Data Availability

The data in the current paper are publicly available since this a meta-analysis conducted on the basis of the cited literature.

## Abbreviations

COVID-19: 2019 Coronavirus disease
RR: Risk ratio
CI: Confidence inerval
SARS-CoV-2: Severe acute respiratory syndrome coronavirus 2
WHO: World Health Organization
LDL-C: Low density lipoprotein cholesterol
TC: Total cholesterol
LDL: Low density lipoprotein
oxLDL: Oxidized low density lipoprotein
HDL-C: High density lipoprotein cholesterol
HDL: High density lipoprotein
NF-κB: Nuclear factor kappa-B
SAA: Serum amyloid A
CRP: C-reactive protein
CMV: Cytomegalovirus
IL-1: Interleukin 1
NLRP3: Nucleotide-binding oligomerization domain-, leucine-rich repeat- and pyrin domain-containing 3
IL-6: Interleukin 6
