# Supplementary material for: Association of dyslipidemia with the severity and mortality of coronavirus disease 2019 (COVID-19): a meta-analysis
Source: Virol J. 2021 Jul 27;18:157. doi: 10.1186/s12985-021-01604-1 (PMC8314261; doi:10.1186/s12985-021-01604-1)
Supplement: Supplementary file 1 — Additional file 1: Table SI: Quality assessment of included studies (cohort studies) [file 12985_2021_1604_MOESM1_ESM.docx]

Table SI: Quality assessment of included studies (cohort studies)

| Author | Selection | Comparability | Outcome | Quality score(0-9) |
| --- | --- | --- | --- | --- |
| Maeda 2020 [8] | 4 | 1 | 2 | 7 |
| Ferguson 2020 [9] | 4 | 1 | 2 | 7 |
| Zhang 2020 [10] | 4 | 1 | 2 | 7 |
| To 2020 [11] | 4 | 1 | 2 | 7 |
| Rastrelli 2020 [12] | 4 | 1 | 3 | 8 |
| Almazeedi 2020 [13] | 4 | 2 | 3 | 9 |
| Chen 2020 [14] | 4 | 1 | 2 | 7 |
| Jurado 2020 [15] | 4 | 1 | 2 | 7 |
| Wu 2020 [16] | 4 | 1 | 2 | 7 |
| Gidari 2020 [18] | 4 | 1 | 2 | 7 |
| Zhang 2020 [19] | 4 | 2 | 3 | 9 |
| de la Rica 2020 [20] | 4 | 1 | 2 | 7 |
| Simonnet 2020 [21] | 4 | 2 | 2 | 8 |
| Petrilli 2020 [22] | 4 | 2 | 3 | 9 |
| Chang 2020 [24] | 4 | 1 | 3 | 8 |
| Kong 2020 [25] | 4 | 1 | 2 | 7 |
| Lodigiani 2020 [26] | 4 | 1 | 3 | 8 |
| Khalil 2020 [27] | 4 | 1 | 3 | 8 |
| Gayam 2020 [28] | 4 | 1 | 3 | 8 |
| Goicoechea 2020 [29] | 4 | 1 | 3 | 8 |
| Santos 2020 [30] | 4 | 2 | 3 | 9 |
| Wang 2020 [31] | 4 | 2 | 3 | 9 |
| Ferrando 2020 [32] | 4 | 1 | 3 | 8 |
| Hwang 2020 [33] | 4 | 2 | 3 | 9 |
| Smith 2020 [34] | 4 | 2 | 3 | 9 |
| Grasselli 2020 [35] | 4 | 2 | 3 | 9 |
