# Supplementary material for: Association of dyslipidemia with the severity and mortality of coronavirus disease 2019 (COVID-19): a meta-analysis
Source: Virol J. 2021 Jul 27;18:157. doi: 10.1186/s12985-021-01604-1 (PMC8314261; doi:10.1186/s12985-021-01604-1)
Supplement: Supplementary file 2 — Additional file 2: Table SII: Quality assessment of included studies (case–control studies) [file 12985_2021_1604_MOESM2_ESM.docx]

Table SII: Quality assessment of included studies (case-control studies)

| Author | Selection | Comparability | Exposure | Quality score(0-9) |
| --- | --- | --- | --- | --- |
| Urra et al 2020 [17] | 3 | 1 | 3 | 7 |
| Li et al 2020 [23] | 3 | 2 | 3 | 8 |
