# Supplementary material for: Association of dyslipidemia with the severity and mortality of coronavirus disease 2019 (COVID-19): a meta-analysis
Source: Virol J. 2021 Jul 27;18:157. doi: 10.1186/s12985-021-01604-1 (PMC8314261; doi:10.1186/s12985-021-01604-1)
Supplement: Supplementary file 3 — Additional file 3: Figure S1: Sensitivity analysis demonstrating the association of dyslipidemia with COVID-19 mortality. [file 12985_2021_1604_MOESM3_ESM.docx]

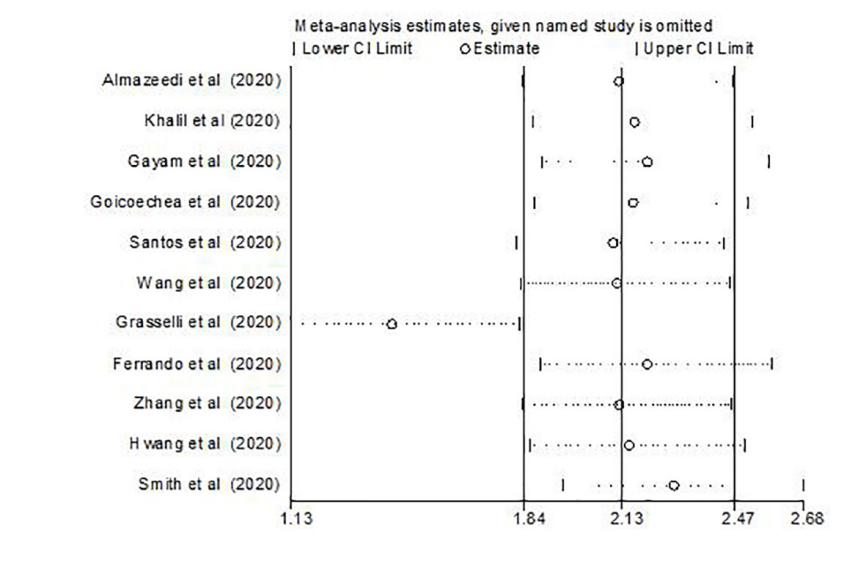


Figure S1: Sensitivity analysis demonstrating the association of dyslipidemia with COVID-19 mortality. Abbreviations: COVID-19, coronavirus disease; CI, confidence interval
